# 2024 Lasker Award Recipient Zhijian Chen elucidates how DNA stimulates immunity

**DOI:** 10.1172/JCI186104

**Published:** 2024-09-19

**Authors:** Amy B. Heimberger

**Affiliations:** Department of Neurological Surgery, Malnati Brain Tumor Institute, Robert H. Lurie Comprehensive Cancer Center, Feinberg School of Medicine, Northwestern University, Chicago, Illinois, USA.

## The discovery of the cGAS/STING pathway

**Figure 1:**
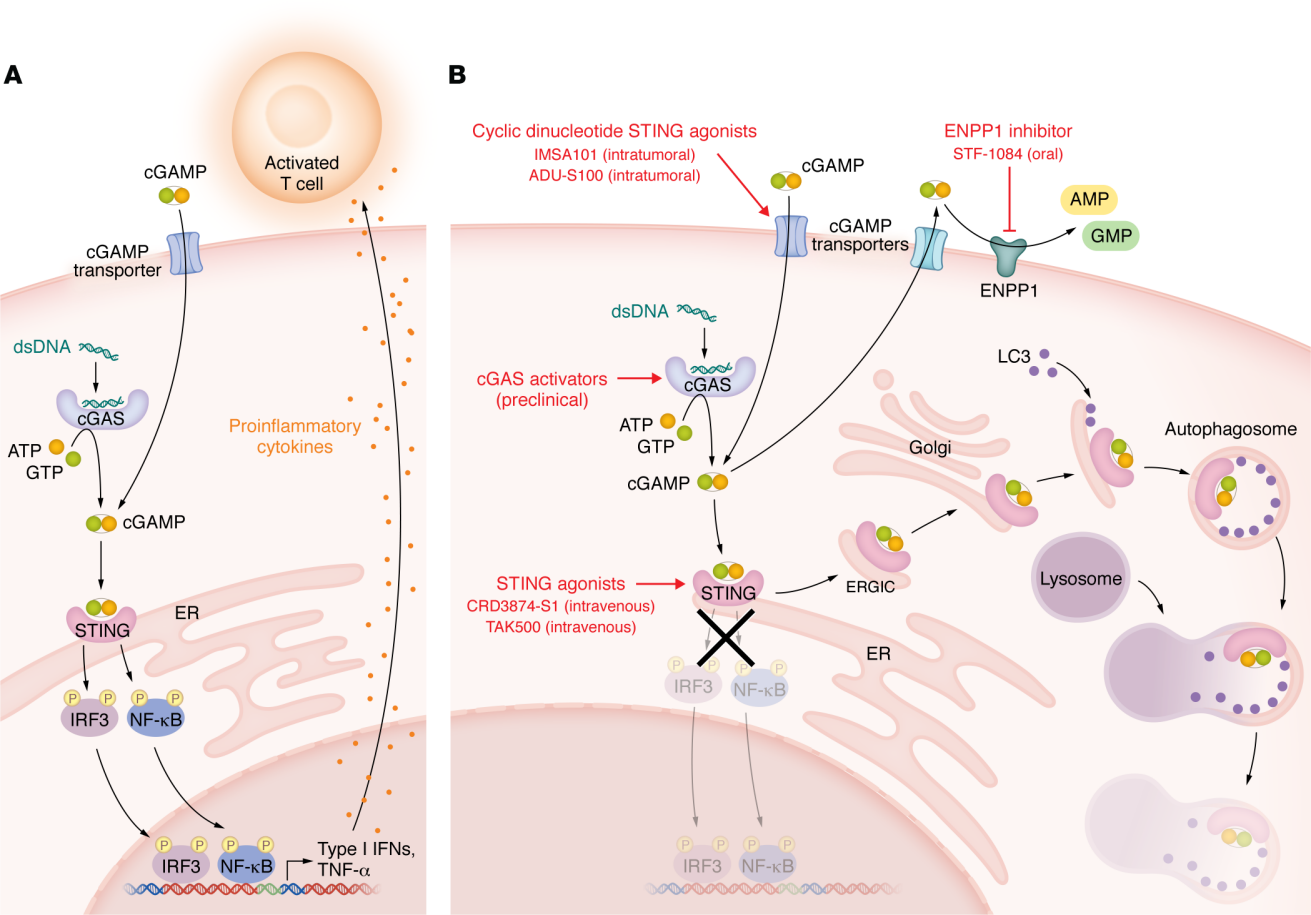
cGAS/STING signaling and therapeutic targeting. (**A**) Activation of the cGAS/STING pathway can be triggered either through the importation of cGAMP or the presence of double-stranded DNA (dsDNA). Activated cyclic GMP-AMP synthase (cGAS) uses ATP and GTP to catalyze the formation of cGAMP that binds to STING localized on the ER membrane. STING then recruits TBK1, which activates/phosphorylates IRF3 and NF-κB, which translocates to the nucleus, thereby inducing the transcriptional activation of proinflammatory interferon responses. (**B**) A variety of therapeutics are being evaluated in clinical trials, such as cyclic dinucleotides or STING agonists that can trigger the activation of cGAS/STING pathway. The STING signal is terminated (denoted by the X) when activated STING in the ER-Golgi compartment binds to LC3 on the autophagy membrane, leading to the degradation of STING and the associated cytoplasmic DNA in the autolysosome. Excess intracellular cGAMP is transported out of the cell and hydrolyzed by ectonucleotide pyrophosphatase phosphodiesterase 1 (ENPP1) to yield AMP and GMP. ENPP1 inhibitors maintain proinflammatory cGAMP concentrations. ERGIC; endoplasmic-reticulum-Golgi intermediate compartment.

Cytoplasmic DNA triggers a danger signal response that has evolved to detect and eradicate infectious organisms. This year’s Albert Lasker Basic Medical Research Award to Dr. Zhijian “James” Chen, Professor of Molecular Biology at UT Southwestern Medical Center, recognizes his contributions to determining the mechanism of how DNA is detected and the connection to the downstream signaling cascade that is essential for initiating an immune response. The field had established that DNA triggers proinflammatory IFN responses through the stimulator of IFN genes (STING) pathway (1) and its phosphorylation of IFN regulatory factor 3 (IRF3) — the transducer of IFN gene transcription (2). Prior studies showed that the multipass transmembrane STING protein functions as a direct innate immune sensor of bacterial elaborated cyclic diguanylate monophosphate (c-di-GMP) and cyclic diadenylate monophosphate (c-di-AMP) (3). Several DNA-sensing pattern-recognition receptors, including AIM2-related protein, IFI16, and DDX41, had previously been linked to STING activation. However, it was the Chen laboratory that discovered that the nucleotidyl transferase cyclic GMP-AMP synthase (cGAS) produces a second-messenger cyclic GMP-AMP (cGAMP) that binds to STING as the principal pathway inducing IFN (Figure 1) (4, 5). Using a combination of mass spectrometry and protein purification strategies, cGAS was identified, and it was shown to bind to DNA and catalyze the synthesis ,of cGAMP from ATP and GTP. cGAMP binds to STING with high affinity, causing extensive ligand-induced conformational changes (6).

## cGAS regulation

During steady-state conditions cGAS is tethered in the nuclear compartment. Under conditions of DNA instability, such as DNA damage, or mitosis, genomic DNA may form micronuclei that interact with cGAS (7). The mechanism for how cGAS was blocked from associating with chromatin during mitosis was unknown. The Chen laboratory showed that during mitosis, cGAS is hyperphosphorylated at its N-terminus, blocking its ability to sense nuclear chromatin but not mitochondrial DNA. Furthermore, oligomerization of chromatin-bound cGAS, which is required for its activation, is prevented. Together, these mechanisms ensure that cGAS remains inactive when associated with chromatin during mitosis (8). Nucleases in the extracellular, cytoplasmic, and endosomal compartments also protect against chronic responses to nucleic acids. For downstream signal transduction to occur in the cytoplasm, cGAS is released from the nuclear tethering through a nuclear export signal (9).

## cGAS/STING signal termination

Activated STING in the endoplasmic reticulum–Golgi compartment binds to LC3 on the autophagy membrane, leading to degradation of STING and termination of the activation signal. This degradation process removes cytoplasmic DNA regardless of its origin through enzymatic destruction in the autolysosome. The fate of intracellular cGAMP differs across cell types, but, in general, excess cGAMP is transported out of cells and hydrolyzed by either soluble or membrane-bound ectonucleotide pyrophosphatase phosphodiesterase 1 (ENPP1) to yield AMP and GMP in the extracellular space (10). When the production and export of cGAMP exceeds the catabolic capacity of ENPP1, cGAMP is transferred into adjacent cells through specific transporters. In tumors, cGAMP can be transported between adjoining cells through gap junctions (11). cGAMP can also enter surrounding cells through specialized cell-surface importers and the fusion of extracellular vesicles. In this context, extracellular cGAMP acts as an immunotransmitter modulating local antitumor immunity.

## Discovery of the cGAS/STING pathway

### Autoimmunity.

Accumulation of endogenously derived DNA in the cytosol can trigger autoimmune disorders (12). Chronically elevated levels of IFN are detected in a subset of patients with systemic lupus erythematosus. Human genetic variation in gene encoding proteins critical for nucleic acid metabolism or DNases is associated with the activation of cGAS in several autommune conditions.

### Neurodegeneration.

Accumulation of aggregated misfolded proteins in neurons and glia is a pathological hallmark of many neurodegenerative diseases. These proteins trigger a stress response, including IFN, which has neurocytotoxic effects. Patients with amyotrophic lateral sclerosis have high levels of IFN and cGAMP in the spinal cord (13). In mice that lack mitochondrial-protective proteins cellular stress induces mitochondrial damage and activation of the cGAS/STING pathway (14). NAD^+^ supplementation or melatonin can reduce neuroinflammation in murine models of neurodegeneration.

### Infection.

Given broad DNA ligand specificity, cGAS is activated by most DNA-containing viruses. For RNA viruses such as HIV, cGAS is activated by Y-form DNA intermediates (15). SARS-CoV-2 activates cGAS by inducing mitochondrial damage, micronuclei generation, and cell fusion, which leads to the nuclear export of chromatin DNA into the cytoplasm (16). Accordingly, in COVID-19, cGAS/STING activation contributes to both lung inflammation and skin manifestations through the induction of endothelial cell death and IFN production. In the brain, microglia and immune cells undergo apoptosis in response to HSV-1 infection that is dependent on cGAS/STING pathway activation as a function of the viral load (17), indicating an activation-induced apoptosis program that downregulates local brain immune responses.

### Oncology.

Therapeutic approaches to modulate the cGAS/STING pathway are a particular focus for oncology. STING expression and the propensity to generate IFN are critical for generating anticancer immune responses. In this context, STING triggers antigen presentation and T cell priming and activation, facilitates immune infiltration into the tumor microenvironment, and promotes immunologically mediated tumor cytotoxicity (18). STING agonists can directly elicit cancer cell death, and chemotherapeutics can stimulate the cGAS/STING pathway. In addition, any agent or strategy that induces DNA damage, such as radiation, initiates pathway activation.

## cGAS/STING pathway in immune checkpoint inhibitor response

Defective mismatch repair (dMMR) in tumors is associated with neoantigen generation (19) and activation of the cGAS/STING pathway. In 50% of dMMR tumors, loss of the MutLα subunit of MLH1 results in unrestrained DNA excision by Exo1, which causes increased single-strand DNA formation, replication protein A (RPA) exhaustion, DNA breaks, and aberrant DNA repair intermediates (20). Ultimately, this generates chromosomal abnormalities and the release of nuclear DNA into the cytoplasm, thus activating the cGAS/STING pathway (20) and predisposing responses to immunotherapy. Checkpoint kinase 2 (Chek2) mediates therapeutic resistance to CD8^+^ T cell recognition, and both genetic depletion or pharmacologic inhibition of Chek2 increase survival in combination with immune checkpoint blockade through STING activation in preclinical glioma models (21). The activation of STING increases the expression of immune checkpoint proteins across many cancer types, and the combination of a STING agonist with immune checkpoint blockade is typically additive to monotherapeutic efficacy (22).

## The race for therapeutic modulation

### Pathway activation.

Enhancing the proinflammatory IFN response can be beneficial in the setting of cancer or viral infections, and IFN-α is used as an adjunctive therapy for some types of cancer and chronic infections. In oncology, one of the first STING agonists, ADU-S100, was evaluated in a phase I study of patients with solid cancers (*n* = 47) treated with weekly intratumoral injections. The injected lesion size was stable or decreased in size in 94% of injected lesions, and a maximum tolerated dose was not reached (23). However, the follow-up phase Ib study of ADU-S100 in combination with anti–PD-1 only demonstrated a response rate of 10% (24), precluding advancement to later-stage clinical trials. Confounders for response, such as determining epigenetic modifications of the STING promoter that silence the STING pathway (25) or the degree of myeloid infiltration, were not assessed.

There are currently four open clinical trials of STING agonists in cancer, including a small-molecule agonist, CRD3874-S1, for sarcoma and Merkel cell carcinoma (NCT06021626); IMSA101 in solid malignancies (NCT05846659; NCT05846646); and the immune cell–targeting drug conjugate, TAK-500 (NCT05070247). The IMSA101 compound, developed by Dr. Chen, is an intratumoral administered agent. This route of administration may limit the number of doses that can be administered and the types of malignancies that can be treated. However, selective delivery to the tumor site may reduce unwanted side effects, such as nonspecific systemic inflammation and the risk of autoimmunity. In contrast, TAK-500 is delivered systemically enabling multidosing. There are no clinical trials directly targeting cGAS, but preclinical efforts are underway. Antibody-STING conjugates have been devised, and preclinical models have shown that systemic administration is well tolerated and can exert antitumor efficacy in murine models (26). A caveat of targeting STING to the tumor cell is that some malignancies have epigenetic silencing of the STING promoter (25). In contrast, some cancers, such as H3.3-G34R gliomas, may have enhanced susceptibility to DNA damage leading to the accumulation of extrachromosomal DNA and thereby increased sensitivity to STING agonists (27). Since STING activation can induce apoptosis in T cells, STING agonists must be titrated to maximize the biological effect.

### Targeting ENPP1 or cGAMP transport.

Tumor cells upregulate ENPP1 on their surface as an immune evasion mechanism (28). High ENPP1 expression is associated with worse prognosis and response to therapy, likely due to low extracellular cGAMP levels coupled with high levels of immune suppressive adenosine. Since secreted cGAMP activates the immune system to reduce tumor growth and spreading, extending the lifespan of extracellular cGAMP by targeting ENPP1 is a therapeutic strategy. STF-1084 can inhibit ENPP1 and improves the response to radiation therapy in preclinical models of cancer (10). The ENPP1 inhibitor SR-8541A is in phase I testing in patients with solid cancers (NCT06063681). Other cGAMP targeting approaches include compounds that block gap junctions (29). The cell type–specific expression of cGAMP importers and differential responsiveness to extracellular cGAMP may provide future opportunities for targeted therapy.

### Pathway inhibition.

In autoimmune disorders, attenuation of IFN activity might improve outcomes. A variety of compounds can inhibit this pathway, but most are only useful in vitro. Whether long-term inhibition of the cGAS/STING pathway could potentially increase susceptibility to infection or cancer is unknown. Given the pathogen-free environments of murine mouse models and the differences in cancer predisposition between mice and humans, these long-term side effects will need to be monitored in humans.

## Concluding remarks

Despite its recent discovery in 2013, there has been remarkable progress in delineating the mechanistic role of the DNA sensor cGAS, its partnership with STING, and its role in inflammation across a wide spectrum of diseases. Given the profound impact of cGAS/STING spanning multiple disciplines, the Lasker Foundation recognizes Dr. Chen with the 2024 Albert Lasker Basic Medical Research Award because his laboratory has been instrumental in identifying the role of cGAS/STING pathway and the rapid development of therapeutics that target this pathway. Improvements in cGAMP detection methods and the evaluation of response to therapies designed to enhance or attenuate the cGAS/STING pathway will further clarify the role of this pathway in human diseases. Whether cGAS, cGAMP, or STING will be the pivotal therapeutic target associated with the fewest side effects remains to be clarified. A more refined mechanistic understanding of how DNA ligands, including mitochondrial DNA, trigger cGAS in autoinflammatory, autoimmune, and degenerative diseases may further inform therapeutic modulation strategies upstream of cGAS.

## Version 1 09/19/2024

Electronic publication

## Version 2 09/25/2024

Electronic publication
